# Influence of the time interval from diagnosis to treatment on survival for early-stage liver cancer

**DOI:** 10.1371/journal.pone.0199532

**Published:** 2018-06-22

**Authors:** Wen-Chen Tsai, Pei-Tseng Kung, Yueh-Hsin Wang, Wei-Yin Kuo, Ya-Hsin Li

**Affiliations:** 1 Department of Health Services Administration, China Medical University, Taichung, Taiwan; 2 Department of Healthcare Administration, Asia University, Taichung, Taiwan; 3 Department of Health Policy and Management, Chung Shan Medical University, Chung Shan Medical University Hospital, Taichung, Taiwan; Institut national de la recherche scientifique, CANADA

## Abstract

**Objectives:**

Liver cancer is the fifth most common cancer in men and the ninth most common cancer in women, and the WHO expects that there will be 1,341,344 cases in 2034 worldwide. Liver cancer also has the second-highest cancer death rate, accounting for 7% of all cancers. The study is going to explore the relationship between time interval from diagnosis to treatment and survival status of early-stage liver cancer patients.

**Materials and methods:**

This is a retrospective cohort study using the national database from Taiwan. The datasets include the Taiwan Cancer Registry Database (TCR), the National Health Insurance Research Database (NHIRD), and the National Registry of Deaths. The target population for the study was patients newly diagnosed with stage I and stage II liver cancer between the years 2004 and 2010. Total of 26,038 cases were included in the study. Except descriptive analysis, the relationship between patient characteristics and the time interval from diagnosis to treatment was examined by chi-square tests. In addition, modified Cox proportional hazard models were used to analyze the hazard ratio of patient death with various treatment delay durations.

**Results:**

There were 20,430 patients (78.46%) who received treatment less than 30 days after diagnosis, while 2,674 patients (10.27%) received treatment between 31 and 60 days after diagnosis, and 2,068 patients (7.94%) received treatment between 61 and 180 days after diagnosis, and 866 patients (3.33%) who received treatment 181 days after diagnosis. Those treated more than 181 days and 61–180 days after diagnosis had a 1.68-fold increased risk of death (95% confidence interval: 1.50–1.88) and a 1.39-fold increased risk of death (95% confidence interval: 1.31–1.17), respectively. Being male, being elderly, having a higher CCI level, and being treated in a hospital with a low service volume were factors associated with a poorer prognosis.

**Conclusion:**

Overall, this study utilized a national cohort to conclude that for early-stage liver cancer patients, a longer the time interval from diagnosis to treatment results in a lower survival rate.

## Introduction

Liver cancer is the fifth most common cancer in men and the ninth most common cancer in women, and the WHO expects that there will be 1,341,344 cases in 2034 worldwide. Liver cancer also has the second-highest cancer death rate, accounting for 7% of all cancers [1]. The disease burden is highest in areas with endemic HBV infection, such as in eastern and south-eastern Asia, with incidence rates of over 30 per 100,000 individuals.

Due to the complexity of cancer, physicians arrange the treatment schedule according to the patients’ individual physical and psychological health status. Cancer treatment is a long and complex process. Many studies have concluded that patient survival rates are significantly higher in patients who were diagnosed early and received immediate treatment [2–5]. However, liver cancer presents no significant symptoms in the early stages, and patients usually see a physician when the tumor size has increased or comorbidities such as liver cirrhosis occur, which may be too late for treatment. Many previous studies have discussed liver cancer prevention and treatment methods. According to the treatment guidelines from the American Cancer Society, the main treatments for liver cancer are surgery, hepatic arterial embolization, electrocauterization, molecular-targeted therapy, and radiation therapy, determined according to the patients’ specific health status and situation.

Many previous studies concluded that treatment delay may stem from the patient or the physician in numerous countries. A delay in starting or a change in the treatment may affect the therapeutic outcome [2,3,6]. One study found that a treatment delay will significantly lower liver cancer patient survival rate (HR: 0.50; 95% CI: 0.30–0.84) [2]. Another study concluded that when patients experienced a treatment delay of longer than 3 months, the survival rate decreased significantly (RR: 3.67; p = 0.002) and could even lead to tumor deterioration [3].

Many factors can cause treatment delays, including patient awareness of symptom severity, lower economic status, alternative treatment methods, fear of cancer treatment, lack of comprehensive knowledge about cancer treatment, and fear of judgement from society [7–11]. However, the health system plays one of the most important roles in potentiating treatment delay. Whether under the private health insurance system (health management organization (HMO)/ preferred provider organization (PPO)) in the U.S. or the National Health Services system (NHS) in the UK, the strict referral system may result in a longer treatment delay, and a long delay is associated with an unfavorable outcome [12–14]. However, there is no referral system in Taiwan. Patients can visit a specialist physician or medical center directly. With respect to catastrophic illness patients such as cancer, stroke, type I diabetes, or dialysis, the Ministry of Health Insurance in Taiwan provides a catastrophic illness card for them. The patients with a catastrophic illness card can receive a waiver for the copayment for outpatient and inpatient services. For rural area residents, the National Health Insurance Administration in Taiwan provides mobile medicine program and Integrated Delivery System to increase the patient’s access to medical care. Therefore, treatment delay due to referral time or financial barriers should not occur often in Taiwan. However, some studies still found that cancer patients did not receive prompt treatment upon diagnosis [15, 16].

There are no related studies that investigate the influence of the time interval from diagnosis to treatment on survival for early-stage liver cancer. Therefore, this study aims to evaluate the influence of the time interval from diagnosis to treatment for early-stage liver cancer patients using national cancer datasets, national health insurance datasets, and death report data.

## Research methods

### Data resources

This is a retrospective cohort study using the national database from Taiwan. The datasets include the Taiwan Cancer Registry Database (TCR), the National Health Insurance Research Database (NHIRD), and the National Registry of Deaths. All newly diagnosed malignant cancers from hospitals with 50 or more beds have been reported to the TCR since 1979. After the passing of the Cancer Control Act in 2003, the data quality and completeness of the cancer registry database has improved significantly. The quality of the TCR data was confirmed by previous studies [17, 18]. The NHIRD contains comprehensive inpatient and outpatient health care information for over 99% of Taiwan’s population. The quality and accuracy of the NHIRD has also been validated in previous studies [19, 20]. This study was approved by the Institutional Review Board of the China Medical University Hospital.

### Participant selection

The target population for the study was patients newly diagnosed with stage I and stage II hepatic carcinomas between the years 2004 and 2010 (International Classification of Diseases, Oncology, 3rd edition [ICD-O-3] codes: C220). A total of 31,427 cases were extracted as the parent group. After excluding no treatment data in the NHIRD database (n = 1,089), multiple cancers (n = 866), solely palliative care cases (n = 334), and cases with incomplete data in the NHIRD and TCRD (n = 3,100), the final study population comprised 26,038 cases.

### Description of variables

The time interval from diagnosis to treatment was defined as the time interval from the date of the biopsy that confirmed malignancy to the date of the patient's first treatment (surgery, radiotherapy, or chemotherapy). After discussion with the clinical physicians, the time intervals were divided into 4 groups: “within 30 days”, “31–60 days”, “61–180 days”, and “more than 181 days”. The subject’s urbanization levels were scored according to their residency before the cancer diagnosis and ranged from highly developed urban cities (level 1) to remote districts (level 7) [21]. The degree of comorbidity was categorized into four levels according to the Charlson Comorbidity Index (CCI) as modified by Deyo [22]. The study used inpatient and outpatient data from the previous 2 years to calculate the CCI. Patients with catastrophic illness card defined as the included cancer patients with any other catastrophic illness (except cancer). The primary hospital services volume was divided into low, medium and high categories based on the quartile. Other variables included patient gender, age, monthly salary, hospital tier (medical centers, regional hospitals, and district hospitals), and hospital ownership (public and private).

### Statistical analysis

All statistical analyses for this study were performed using SAS software, version 9.2 (SAS Institute Inc., Cary, NC). The purpose of this study is to assess the influence of the time interval from diagnosis to treatment on patient survival rates for stage I and stage II liver cancer. The study used descriptive analysis to present basic patient information (gender, age at diagnosis), economic status (salary level), residence status (urbanization levels), health status (CCI, cancer stage, catastrophic illness status), primary hospital information (hospital tier, ownership, and services volume), and the distribution of the time interval from diagnosis to treatment. The relationship between patient characteristics and the time interval from diagnosis to treatment was examined by chi-square tests. In addition, modified Cox proportional hazard models were used to analyze the hazard ratio of patient death with various treatment delay durations after adjusting for age, sex, and other variables. Finally, an estimation of survival time was calculated by the Kaplan-Meier method and stratified by various tumor stages in order to investigate the influences of diagnosis-to-treatment time interval on the survival of liver cancer patients. All tests in the study were two-tailed, and a P value of < 0.05 was regarded as statistically significant.

## Results

The average observation time for the total of 26,038 patients was39.25±25.85 months, and the average patient age was 63.37±12.12 years. There were 20,430 patients (78.46%) who received treatment less than 30 days after diagnosis, 2,674 patients (10.27%) who received treatment between 31 and 60 days after diagnosis, 2,068 patients (7.94%) who received treatment between 61 and 180 days after diagnosis, and 866 patients (3.33%) who received treatment 181 days after diagnosis. The average follow-up time for the four duration groups was 40.48±26.23 months, 36.48±23.89 months, 33.00±23.57 months, and 33.75±24.31 months (P < 0.001), respectively. A trend emerged, showing that a longer time interval from diagnosis to treatment corresponded to a shorter follow-up time. Of all patients, 61.01% (N = 15,886) were classified as stage I, while 38.99% of patients (N = 10,152) were in stage II. Additional descriptive data are presented in Table 1.

**Table 1:** Descriptive statistics of liver cancer patients with different time intervals from diagnosis to treatment.

Variables		Total		Interval from cancer diagnosis to treatment								P value
				≤ 30 days		31~60 days		61~180 days		≥ 181 days		
		N	%	N	%	N	%	N	%	N	%	
**Total number**		28,036	100.00	21,123	75.34	2,762	9.85	2,124	7.58	2,027	7.23	-
**Death or alive**												<0.001
	Alive	12,062	43.02	9,911	82.17	1,109	9.19	618	5.12	424	3.52	
	Death	15,974	56.98	11,212	70.19	1,653	10.35	1,506	9.43	1,603	10.04	
**Average follow-up months (m, sd)**		38.24	25.96	39.94	26.32	35.78	24.06	32.38	23.62	29.99	24.21	<0.001
**Gender**												<0.001
	Female	8,908	31.77	6,410	71.96	996	11.18	743	8.34	759	8.52	
	Male	19,128	68.23	14,713	76.92	1,766	9.23	1,381	7.22	1,268	6.63	
**Age**												<0.001
	≤ 44	1,904	6.79	1,557	81.78	132	6.93	112	5.88	103	5.41	
	45~54	4,417	15.75	3,433	77.72	391	8.85	327	7.40	266	6.02	
	55~64	7,471	26.65	5,734	76.75	724	9.69	562	7.52	451	6.04	
	65~74	8,761	31.25	6,613	75.48	919	10.49	656	7.49	573	6.54	
	≥ 75	5,483	19.56	3,786	69.05	596	10.87	467	8.52	634	11.56	
**Mean age (m, sd)**		63.61	12.19	63.03	12.17	65.00	11.55	64.59	12.04	66.69	12.77	<0.001
**Monthly salary**												<0.001
	Low-income	272	0.97	189	69.49	33	12.13	24	8.82	26	9.56	
	≤ 17280	945	3.37	703	74.39	92	9.74	76	8.04	74	7.83	
	17281~22800	15,011	53.54	11,166	74.39	1,476	9.83	1,168	7.78	1,201	8.00	
	22801~28800	4,588	16.36	3,390	73.89	521	11.36	366	7.98	311	6.78	
	28801~36300	1,884	6.72	1,463	77.65	174	9.24	141	7.48	106	5.63	
	36301~45800	2,471	8.81	1,949	78.87	223	9.02	159	6.43	140	5.67	
	≥ 45801	2,865	10.22	2,263	78.99	243	8.48	190	6.63	169	5.90	
**Urbanization**												<0.001
	Level 1	6,951	24.79	5,367	77.21	635	9.14	488	7.02	461	6.63	
	Level 2	7,937	28.31	5,943	74.88	834	10.51	611	7.70	549	6.92	
	Level 3	4,011	14.31	3,044	75.89	392	9.77	297	7.40	278	6.93	
	Level 4	4,831	17.23	3,611	74.75	472	9.77	382	7.91	366	7.58	
	Level 5	1,179	4.21	848	71.93	122	10.35	89	7.55	120	10.18	
	Level 6	1,666	5.94	1,184	71.07	180	10.80	146	8.76	156	9.36	
	Level 7	1,461	5.21	1,126	77.07	127	8.69	111	7.60	97	6.64	
**CCI score**												<0.001
	≤ 3	17,861	63.71	13,821	77.38	1,686	9.44	1,229	6.88	1,125	6.30	
	4~6	7,356	26.24	5,311	72.20	781	10.62	633	8.61	631	8.58	
	≥ 7	2,819	10.05	1,991	70.63	295	10.46	262	9.29	271	9.61	
**Catastrophic illness**												<0.001
	No	25,038	89.31	19,093	76.26	2,408	9.62	1,824	7.28	1,713	6.84	
	Yes	2,998	10.69	2,030	67.71	354	11.81	300	10.01	314	10.47	
**Cancer stage**												<0.001
	Stage I	17,048	60.81	13,362	78.38	1,529	8.97	1,084	6.36	1,073	6.29	
	Stage II	10,988	39.19	7,761	70.63	1,233	11.22	1,040	9.46	954	8.68	
**Hospital level**												<0.001
	Medical centers	19,393	69.17	14,846	76.55	1,943	10.02	1,436	7.40	1,168	6.02	
	Regional hospitals	8,128	28.99	5,926	72.91	793	9.76	646	7.95	763	9.39	
	District hospitals	515	1.84	351	68.16	26	5.05	42	8.16	96	18.64	
**Hospital ownership**												<0.001
	Public	9,344	33.33	7,274	77.85	816	8.73	677	7.25	577	6.18	
	Private	18,692	66.67	13,849	74.09	1,946	10.41	1,447	7.74	1,450	7.76	
**Hospital services volume**												<0.001
	Low	6,101	21.76	4,441	72.79	511	8.38	512	8.39	637	10.44	
	Middle	14,226	50.74	10,778	75.76	1,412	9.93	1,051	7.39	985	6.92	
	High	7,709	27.50	5,904	76.59	839	10.88	561	7.28	405	5.25	

A longer time interval from diagnosis to treatment corresponded to a lower survival rate according to the Cox proportional hazards model (Table 2). When comparing the patients treated within 30 days to those treated between 31 and 60 days, between 61 and 180 days, and after 181 days, the death risk was 1.14-fold (95% CI: 1.08–1.20), 1.43-fold (95% CI: 1.35–1.51), and 1.48-fold (95% CI: 1.37–1.60), respectively. Sex, age, economics status, CCI level, and other critical illnesses, cancer stage, and treatment hospital characteristics were found to be significantly related to the patient survival rate (Table 2).

**Table 2:** Factors associated with overall survival in liver cancer patients.

Variables		Unadjusted		Adjusted			
		HR	P value	HR	95% CI		P value
**Interval from cancer diagnosis to treatment**							
	≤ 30 days (ref.)						
	31~60 days	1.24	<0.001	1.14	1.09	1.20	<0.001
	61~180 days	1.62	<0.001	1.43	1.35	1.51	<0.001
	≥ 181 days	1.95	<0.001	1.61	1.53	1.70	<0.001
**Gender**							
	Female (ref.)						
	Male	0.95	0.003	1.07	1.03	1.11	<0.001
**Age**							
	≤ 44 (ref.)						
	45~54	1.25	<0.001	1.16	1.07	1.26	<0.001
	55~64	1.38	<0.001	1.27	1.18	1.37	<0.001
	65~74	1.67	<0.001	1.50	1.39	1.62	<0.001
	≥ 75	2.46	<0.001	2.13	1.97	2.30	<0.001
**Monthly salary**							
	Low-income (ref.)						
	≤ 17280	0.74	<0.001	0.78	0.66	0.93	0.005
	17281~22800	0.80	0.005	0.78	0.67	0.91	0.001
	22801~28800	0.72	<0.001	0.75	0.64	0.87	<0.001
	28801~36300	0.61	<0.001	0.70	0.60	0.83	<0.001
	36301~45800	0.60	<0.001	0.70	0.59	0.82	<0.001
	≥ 45801	0.55	<0.001	0.63	0.54	0.74	<0.001
**Urbanization level**							
	Level 1 (ref.)						
	Level 2	1.05	0.034	0.98	0.94	1.02	0.369
	Level 3	1.11	<0.001	1.07	1.01	1.13	0.013
	Level 4	1.20	<0.001	1.02	0.97	1.08	0.383
	Level 5	1.23	<0.001	0.99	0.91	1.08	0.868
	Level 6	1.36	<0.001	1.09	1.02	1.17	0.014
	Level 7	1.10	0.015	0.97	0.89	1.04	0.368
**CCI score**							
	≤ 3 (ref.)						
	4~6	1.53	<0.001	1.34	1.29	1.39	<0.001
	≥ 7	2.20	<0.001	1.80	1.72	1.89	<0.001
**Catastrophic Illness**							
	No (ref.)						
	Yes	1.57	<0.001	1.31	1.25	1.38	<0.001
**Cancer stage**							
	Stage I (ref.)						
	Stage II	1.59	<0.001	1.51	1.47	1.56	<0.001
**Hospital level**							
	Medical centers (ref.)						
	Regional hospitals	1.26	<0.001	0.99	0.95	1.03	0.732
	District hospitals	1.61	<0.001	1.07	0.96	1.20	0.211
**Hospital ownership**							
	Public (ref.)						
	Private	1.13	<0.001	1.06	1.03	1.10	0.001
**Hospital services volume**							
	Low (ref.)						
	Middle	0.75	<0.001	0.82	0.78	0.85	<0.001
	High	0.57	<0.001	0.65	0.62	0.69	<0.001

**Fig 1:**
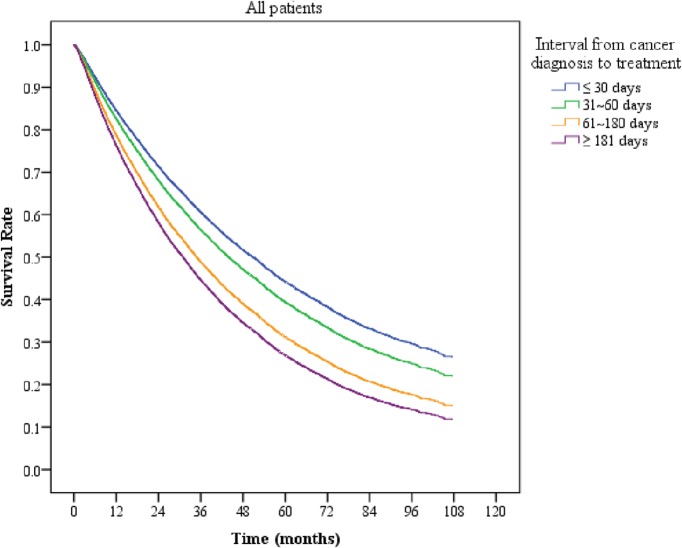
Overall survival curves of liver cancer patients stratified by different time intervals from diagnosis to treatment.

**Fig 2:**
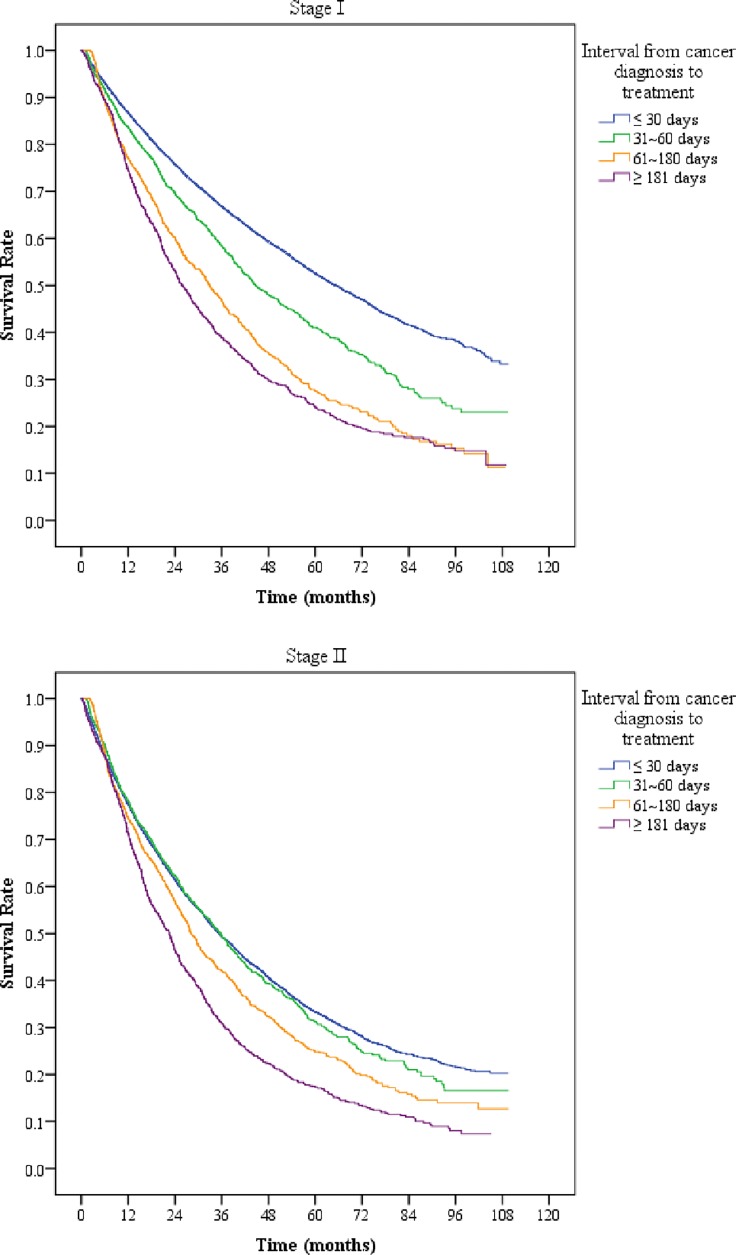
Overall survival curves of liver cancer patients stratified by different time intervals from diagnosis to treatment.

The study stratified patients into stage I and stage II groups. The overall death rate was 50.15% for stage I cancer patients and 67.56% for stage II cancer patients. According to the Kaplan-Meier method, patients treated more than 181 days from diagnosis had the lowest overall survival rate compared to those treated within 30 days (Fig 1). If we stratified the patients according to their initial tumor stage, the time interval from diagnosis to treatment remained a significant prognosticator (Fig 2). For patients with stage I cancer, a longer time interval from diagnosis to treatment corresponded to a lower survival rate. For patients with stage II cancer, the survival rate decreased significantly when the time interval from diagnosis to treatment was longer than 60 days (Fig 2).

The Cox proportional hazards model showcases the prognostic factors for patients with different stages of cancer. In comparison to patients treated within 30 days, a longer time interval from diagnosis to treatment corresponded to a higher death rate. For patients with stage I and stage II cancer, those treated more than 181 days after diagnosis had a 1.68-fold increased risk of death (95% confidence interval: 1.50–1.88) and a 1.39-fold increased risk of death (95% confidence interval: 1.31–1.17), respectively. Being male, being elderly, having a higher CCI level, and being treated in a hospital with a low service volume were factors associated with a poorer prognosis (Table 3). However, some risk factors differed for different cancer stage. Economics status was not related to survival among stage I patients, while patients with a lower economic status had a higher death rate among the stage II patients. The treatment hospital ownership was not related to survival among stage I patients, while those treated in private hospitals had a higher death rate among stage II patients (HR: 1.12; 95% CI: 1.06–1.19).

**Table 3:** Factors associated with overall survival in liver cancer patients under different stage.

Variables		Stage I				Stage II			
		HR	95% CI		P value	HR	95% CI		P value
**Interval from cancer diagnosis to treatment**									
	≤ 30 days (ref.)								
	31~60 days	**1.27**	1.18	1.37	<0.001	**1.01**	0.94	1.09	0.789
	61~180 days	**1.69**	1.57	1.83	<0.001	**1.20**	1.12	1.30	<0.001
	≥ 181 days	**1.83**	1.70	1.98	<0.001	**1.41**	1.31	1.52	<0.001

Control gender, age, monthly salary, urbanization level, CCI score, catastrophic illness, hospital Level, hospital ownership, and hospital services volume

## Discussion

According to the study, only 78.46% of early-stage liver cancer patients received treatment within 30 days after diagnosis. A previous study found that for the early-stage oral cancer patients, 88.67% of patients received treatment within 30 days, which is significantly higher than the percentage of liver cancer patients [16]. This may be due to a lack of obvious symptoms in early-stage cancer patients and the difficulty of clinical diagnosis. According to the AJCC system, stage I cancer patients with a single tumor and no vascular invasion or metastasis, were mainly cirrhosis patients for whom effective treatments were not suitable (only 13–33% of them could receive treatment). These patients may require a longer time to seek out appropriate treatments, increasing the time interval from diagnosis to treatment [23, 24].

This research is a cohort study using national secondary datasets, and the results showed that the risk of death significantly increases with a larger time interval from diagnosis to treatment (Table 1). The results were similar to what was found in previous studies, showing that the survival rate of liver cancer patients decreased with a greater time interval from diagnosis to treatment [2,3].

Although the study did not address all the related factors that affect when cancer patients receive treatment after diagnosis, the results pointed to an association between patient characteristics and the interval time from diagnosis to treatment. The study found that males were more likely to receive treatment within 30 days after diagnosis. This association was consistent with previous studies [25, 26]. We speculated that cultural differences and health insurance policies may affect the patient’s attitude toward seeking treatment. According to the study by Chiou [27], female cancer patients were significantly more likely to shop for outpatient care than males. This might be the reason that female patients have a longer interval time from diagnosis to treatment. In addition, the study found that monthly salary was significantly related to the time interval from diagnosis to treatment, with patients in the lowest salary levels having the longest time interval from diagnosis to treatment. These results were also found in previous studies [25, 26, 28]. In addition, many studies concluded that liver patients of a lower socioeconomic status had a higher death rate when compared to patients with a higher socioeconomic status [29–31]. After the National Health Insurance was implemented in Taiwan in 1994, patient medical accessibility was determined according to their medical needs instead of their income level or residential area [32, 33]. However, the time interval from liver cancer diagnosis to treatment was still different for patients with different monthly salaries, showing that the health inequity problem still exists.

With respect to the levels of urbanization, the study found that those living in level 6 (the aging cities) and level 7 (agricultural cities) had the lowest proportion of patients receiving treatment within 30 days after diagnosis. This may be because these cities have a larger elderly population, and some invasive or aggressive treatments were not suitable for them. In addition, seeking alternative treatments may have increased the interval time from diagnosis to treatment. Similar results can also be found in the study by Bilimoria [34], which reported that an aging population had a higher probability of receiving treatment more than 30 days after diagnosis. Concerning death risk, patients living in level 4 (newly developed cities) and level 7 (agricultural cities) cities had higher death rates than patients living in level 1 cities. The risk factors for developing liver cancer are not only hereditary but also include alcohol consumption, smoking, obesity, diabetes, and family medical history. People living in developing areas or agricultural cities may be more likely to be alcohol users or smokers [35–37], leading to the higher death rate.

The study also found that patients treated in medical centers, private hospitals, and in hospitals with a higher services volume, had a higher probability of receiving treatment within 30 days after diagnosis. A previous study reported the same results [16]. Another study also found that patients receiving treatment or surgery in hospitals with a higher services volume had better outcomes due to a better quality of medical services [38]. In addition, patients with a higher CCI level, those with other critical illnesses, or those with late-stage cancer had a longer time interval from diagnosis to treatment, as well as a higher death rate. Many previous studies concluded that patients with a lower health status, or those with comorbidities, had a higher death rate compared to other patients [39–42].

The definition of time interval from diagnosis to treatment varied in previous studies and can be divided into patient-mediated delay, physician-mediated delay, and delay due to the health care system [43–45]. This research utilized secondary datasets to perform a cohort study. However, the secondary datasets could not conclusively define the reasons for the treatment delay.

Overall, this study utilized a national cohort to conclude that for early-stage liver cancer patients, a longer the time interval from diagnosis to treatment results in a lower survival rate. Thus, encouraging patients receive treatment earlier could increase survival rates. In addition, patient characteristics, such as economics status, health status, and treatment hospital may affect patient survival rate.
